# Association Between sTREM2, an Immune Biomarker of Microglial Activation, and Aging-Related Brain Volume Changes in Community-Dwelling Older Adults: A 7-Year Follow-Up Study

**DOI:** 10.3389/fnagi.2021.665612

**Published:** 2021-04-26

**Authors:** Ryuzo Orihashi, Yoshito Mizoguchi, Yoshiomi Imamura, Shigeto Yamada, Akira Monji

**Affiliations:** ^1^Department of Psychiatry, Faculty of Medicine, Saga University, Saga, Japan; ^2^St. Lucia’s Hospital, Kurume, Japan

**Keywords:** sTREM2, brain volume, cognitive function, MRI, voxel-based morphometry

## Abstract

**Background:**

This study aimed to investigate the association between serum levels of soluble triggering receptor expressed on myeloid cells 2 (sTREM2), a soluble form of an innate immune receptor expressed on the microglia, and brain volume in older adults.

**Methods:**

The survey was conducted twice in Kurokawa-cho, Imari, Saga Prefecture, Japan, among people aged 65 years and older. We collected data from 596 residents. Serum sTREM2 level measurements, brain MRI, Mini-Mental State Examination (MMSE), and clinical dementia rating (CDR) were performed at Time 1 (2009–2011). Follow-up brain MRI, MMSE, and CDR were performed at Time 2 (2016–2017). The interval between Time 1 and Time 2 was approximately 7 years. Sixty-nine participants (16 men, mean age 72.69 ± 3.18 years; 53 women, mean age 72.68 ± 4.64 years) completed this study. We analyzed the correlation between serum sTREM2 levels (Time 1) and brain volume (Time 1, Time 2, and Time 1–Time 2 difference) using voxel-based morphometry implemented with Statistical Parametric Mapping.

**Results:**

Participants in this study had lower MMSE and higher CDR scores 7 years after the baseline evaluation. However, analyses at the cluster level by applying multiple comparison corrections (family wise error; *P* < 0.05) showed no correlation between serum sTREM2 levels and volume of different brain regions, either cross-sectional or longitudinal.

**Conclusion:**

Serum sTREM2 level could not serve as an immune biomarker of aging-related volume changes in brain regions closely related to cognitive function in older adults aged 65 years and above.

## Introduction

Dementia, including Alzheimer’s disease (AD), is a major public health problem globally. Identifying biomarkers that could predict dementia at an early stage will be extremely beneficial. AD pathogenesis is associated with neuroinflammation mainly induced by the activation of microglia in the brain (Haraguchi et al., 2017; Podleśny-Drabiniok et al., 2020). Triggering receptor expressed on myeloid cells 2 (TREM2), a surface receptor of microglial cells, has important roles in microglial functions including phagocytosis and modulation of neuroinflammation (Ulrich and Holtzman, 2016). TREM2 has also been identified as one of the most potent genetic risk factors for AD (Ulrich et al., 2017). TREM2 is released into the extracellular space as a soluble form (sTREM2) and can be detected in the cerebrospinal fluid (CSF) and peripheral blood (Wunderlich et al., 2013; Kleinberger et al., 2014). Several studies reported an association between sTREM2 levels in the CSF or peripheral blood and AD (Hu et al., 2014; Henjum et al., 2016; Heslegrave et al., 2016; Piccio et al., 2016; Suárez-Calvet et al., 2016; Liu et al., 2018). However, to the best of our knowledge, not many studies have analyzed the association between sTREM2 level in peripheral blood and brain volume (Tan et al., 2017). If the association between sTREM2 level in peripheral blood and brain volume is clarified, it will strengthen the evidence that sTREM2 is a biomarker involved in the onset and progression of AD. This study aimed to evaluate serum sTREM2 levels in older adults living in a rural community and examine its relationship with brain volume using MRI. To address this, we designed a prospective cohort study in which healthy older adults without dementia were examined longitudinally for 7 years.

## Materials and Methods

### Participant Characteristics

This was a longitudinal study conducted in Kurokawa-cho, Imari, Saga Prefecture, Japan, among people aged 65 years and above, as reported previously (Nabeta et al., 2014; Matsushima et al., 2015; Imamura et al., 2017; Orihashi et al., 2020). Kurokawa-cho is in northwestern Saga Prefecture and is a rural town somewhat cut-off from urban areas. The area of the town is 26.48 km^2^. As of 2010, the population of Kurokawa-cho was 3253, with 932 people aged 65 years and older (28.7%). The town had 1134 households, and the average number of people per household was 2.87. The main industries are shipbuilding and primary industries.

**FIGURE 1:**
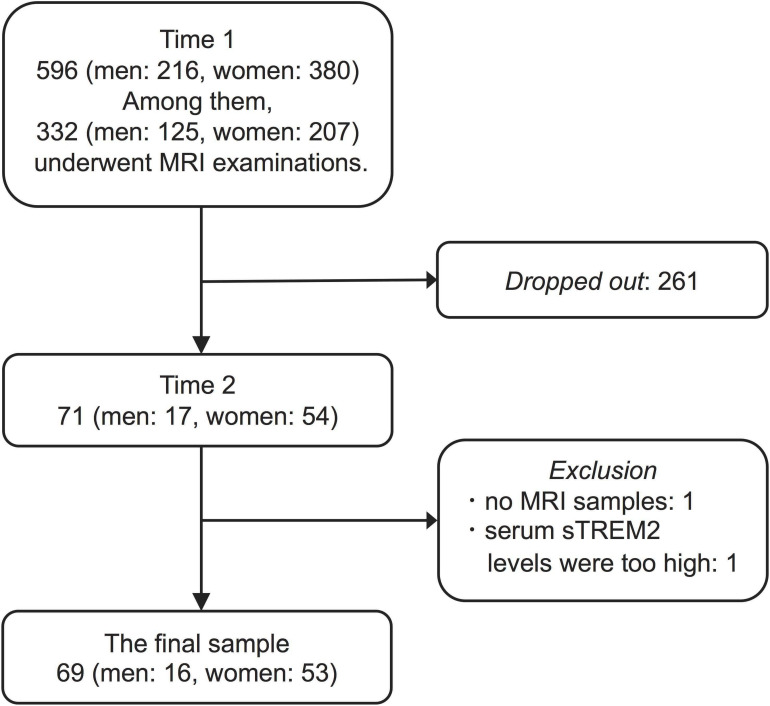
Flow chart depicting selection procedure.

In this study, we collected data from 596 older adults living in the community. These 596 participants comprised 63.9% of the population of Kurokawa-cho over 65 years of age. This survey was conducted twice. First, from October 2009 to March 2011, we conducted a baseline survey that we termed “Time 1.” Second, we conducted from November 2016 to September 2017 (Time 2). Because most of the survey during Time 1 was conducted as a part of the national survey to obtain data to calculate the prevalence of dementia in Japan (Ikejima et al., 2012), not all participants underwent examinations during this period. Specifically, we and Ikejima et al. used the Diagnostic and Statistical Manual of Mental Disorders, third edition-revised, for the diagnosis of dementia with reference to MRI findings. MRI examinations were optional and executed in cases for which it was necessary for further assessment of dementia or the participants themselves requested it. Three hundred thirty-two participants underwent MRI examination and ten were diagnosed with dementia at Time 1. Seven years after conducting the first survey (Time 1), we had notified the investigation of Time 2 to all participants in Time 1 survey. However, only 71 participants (of Time 1) agreed to participate in the investigation of Time 2. Of the 71 participants, none were diagnosed with dementia at Time 1. Thus, 261 participants dropped out arbitrarily between Time 1 and Time 2 (Figure 1).

To select participants for analysis, we excluded two participants with no MRI samples or serum sTREM2 levels higher than three standard deviations from 71 participants. Consequently, we obtained sixty-nine participants (16 men, mean age 72.69 ± 3.18 years; 53 women, mean age 72.68 ± 4.64 years, at Time 1) for analysis.

This study was approved by the Ethics Committee of the Faculty of Medicine, Saga University, and all participants agreed to participate in the study according to the Declaration of Helsinki.

### Cognitive Function Assessment

The Mini-Mental State Examination (MMSE) is a simple screening index that provides an estimate of cognitive function (Folstein et al., 1975). The clinical dementia rating (CDR) is used for dementia evaluation and severity staging (Hughes et al., 1982; Morris, 1993). All participants underwent MMSE and CDR for cognitive function assessment at Time 1 and Time 2.

### Serum Samples

Blood samples for serum sTREM2 levels analysis were collected from participants either between 9:00 and 12:00 (AM) or between 12:00 and 15:00 (PM) during Time 1. On the same day, at Saga University, the samples were centrifuged and the serum was extracted and transferred to a container. The serum samples were immediately stored at −80°C.

### Evaluation of Serum sTREM2 Levels and Other Risk Factors

Serum was thawed at room temperature. All samples were analyzed in duplicate. Serum sTREM2 levels were analyzed using a commercially available human TREM2 ELISA kit (RayBiotech, Norcross, GA, United States) according to the manufacturer’s instructions (Tanaka et al., 2018, 2019; Ohara et al., 2019). The intra-assay coefficient of variation was 10% and the inter-assay coefficient of variation was 12%. The baseline survey also included metabolic status such as body mass index (BMI) and history of diabetes and dyslipidemia. Serum high-sensitivity C-reactive protein (hs-CRP) was analyzed using commercially available ELISA kits (R&D systems, Minneapolis, MN, United States) according to the manufacturer’s instructions. The intra-assay coefficient of variation was 5.5% and the inter-assay coefficient of variation was 6.53%.

### MRI Acquisition

MRI examinations were performed using a 1.5 Tesla device (Excelart Vantage AGV; Canon Medical Systems, Otawara, Japan). Three-dimensional T1-weighted structural images were acquired for each participant using a field echo three-dimensional (FE3D) method (TR, 21 ms; TE, 5.5 ms; flip angle, 20°; field of view, 240 × 240 mm; matrix, 256 × 256; slice thickness, 1.5 mm; number of slices, 124). The examination conditions were kept same for all participants and followed a standardized procedure.

### Statistical Analysis

Participants’ basic data were analyzed and compared using a commercially available statistical package (JMP 14.2.0; SAS Institute, Cary, NC, United States). The mean values were compared using Welch’s *t*-test. Fisher’s exact test was used to compare the prevalence of diabetes and dyslipidemia, and blood collection time. Multiple regression analysis was used to determine the effect of age, sex, hs-CRP, metabolic status, and blood collection time on serum sTREM2 levels. The Wilcoxon signed-rank test was used to compare MMSE and CDR scores at Time 1 and Time 2. Additionally, the serum sTREM2 levels were divided into quartile categories. We used linear mixed-effects models to estimate changes of averaged total brain volumes (gray matter volume and white matter volume) and averaged MMSE scores between Time 1 and Time 2 in each quartile category. Statistical significance was set at *P* < 0.05.

### Preprocessing of the Brain MRI and Longitudinal Voxel-Based Morphometry Analysis

Brain MRI processing and analysis were conducted using voxel-based morphometry (VBM) (Ashburner and Friston, 2000) implemented with Statistical Parametric Mapping (SPM12; Wellcome Department of Cognitive Neurology, London, United Kingdom) in MATLAB R2016a (MathWorks, Natick, MA, United States). We used the same methodology described in a previous study (Orihashi et al., 2020).

T1-weighted MR images were first segmented for gray matter and white matter using the segmentation procedures implemented in SPM12. The diffeomorphic anatomical registration through exponentiated lie algebra (DARTEL) tool described in SPM12 was used on the segmented gray matter and white matter images to construct a template for co-registration across participants (Ashburner and Friston, 2000; Ashburner, 2007). The segmented gray matter and white matter images were co-registered to the final DARTEL template and local volumes were preserved by modulating the image intensity of each voxel by the Jacobian determinants of the deformation fields computed by DARTEL. The registered images were smoothed with a Gaussian kernel with full width at half maximum (FWHM) of 8 mm and transformed into Montreal Neurological Institute (MNI) stereotactic space using affine and non-linear spatial normalization as implemented in SPM12. Preprocessing was conducted by Araya Brain Imaging (Tokyo, Japan).

Gray matter images were used for this analysis. After preparing the Time 1 and Time 2 images, the Time 1–Time 2 difference images were created by subtracting the Time 2 images from the Time 1 images (Ashburner and Ridgway, 2012). Correlation between serum sTREM2 levels at baseline (Time 1) and brain volume (Time 1, Time 2, and Time 1–Time 2 difference) was evaluated using gray matter images and multiple regression design. Men and women were analyzed together, with age, sex, handedness, and hs-CRP levels as covariates. Moreover, total brain volume at Time 1 and Time 2 was used as covariates during the respective time points. The masking toolbox was used to create mask images for analysis, and multiple comparison correction (family wise error) was performed. The initial voxel threshold was set to *P* = 0.001 uncorrected. Clusters were considered significant when they fell below the cluster-corrected P (family wise error) value (=0.05). Thus, analyses at the cluster level were performed to identify significant brain regions. After statistically significant brain regions were determined, the anatomical labels were identified using the automated anatomical labeling corresponding to the space of the MNI standard coordinate system (Tzourio-Mazoyer et al., 2002). Furthermore, using a similar method, analyses were performed on the association between MMSE or CDR scores (Time 1–Time 2 difference) and brain volume (Time 1, Time 2, and Time 1–Time 2 difference).

## Results

### Participant Characteristics, Serum sTREM2 Levels, and MMSE and CDR Scores

There was no significant difference in serum sTREM2 levels between men (344.3 ± 305.4 pg/ml) and women (288.7 ± 277.0 pg/ml) at baseline. Further, the average interval between Time 1 and Time 2 brain MRI examinations was same in men and women. Moreover, no sex differences were observed in the prevalence of other risk factors and blood collection time (Table 1). Multiple regression analysis was performed to assess the association between serum sTREM2 levels and age, sex, hs-CRP, metabolic status, and blood collection time. Result showed that a history of diabetes may be one of the factors affecting serum sTREM2 levels (*P* = 0.03, standard partial regression coefficient = 0.279). Overall, participants’ MMSE scores declined and CDR scores increased from Time 1 to Time 2 (Table 2). Moreover, serum sTREM2 levels did not correlate with changes (i.e., difference between Time 1 and Time 2) in either MMSE (*P* = 0.38) or CDR (*P* = 0.47) scores.

**TABLE 1:** Participant demographics.

	**Overall**	**Men**	**Women**	**Statistical significance**
*N*	69	16	53	
Age (years, Time 1), mean ± SD	72.68 ± 4.32	72.69 ± 3.18	72.68 ± 4.64	ns^a^
sTREM2 (pg/ml, Time 1), mean ± SD	301.6 ± 282.5	344.3 ± 305.4	288.7 ± 277.0	ns^a^
hs-CRP (ng/ml, Time 1), mean ± SD	1199.7 ± 1403.0	1014.3 ± 933.4	1255.7 ± 1519.5	ns^a^
Education (years), mean ± SD	9.90 ± 1.70	10.81 ± 2.10	9.62 ± 1.48	*P* = 0.048^a^
MRI interval (years, Time 1 to Time 2), mean ± SD	6.87 ± 0.63	6.86 ± 0.64	6.88 ± 0.63	ns^a^
BMI (kg/m^2^), mean ± SD	23.90 ± 3.24	23.98 ± 2.73	23.87 ± 3.40	ns^a^
Diabetes, *n* (%)	14 (20.6)	3 (18.8)	11 (21.2)	ns^b^
Dyslipidemia, *n* (%)	26 (38.2)	3 (18.8)	23 (44.2)	ns^b^
Blood collection time, *n* (%)				
AM	35 (50.7)	6 (37.5)	29 (54.7)	ns^b^
PM	34 (49.3)	10 (62.5)	24 (45.3)	

*Missing data: BMI (*N* = 2), Diabetes (*N* = 1), Dyslipidemia (*N* = 1). The blood collection time was defined as “AM” for 9:00 to 12:00 collection and “PM” for 12:00 to 15:00 collection.*

*^*a*^Welch’s *t-*test.*

*^*b*^Fisher’s exact test.*

*ns, not significant; sTREM2, soluble triggering receptor expressed on myeloid cells 2; hs-CRP, high-sensitivity C-reactive protein; BMI, body mass index.*

**TABLE 2:** MMSE and CDR scores at Time 1 and Time 2.

	**Time 1**	**Time 2**	**Statistical significance**
MMSE, mean ± SD	28.42 ± 1.45	26.43 ± 3.64	*P* < 0.0001
**CDR, *n* (%)**			
0	66 (95.7)	62 (89.9)	*P* = 0.029
0.5	3 (4.3)	6 (8.7)	
1		1 (1.4)	

*Wilcoxon signed-rank test.*

*MMSE, Mini-Mental State Examination; CDR, clinical dementia rating.*

### Voxel-Based Morphometry Findings

**FIGURE 2:**
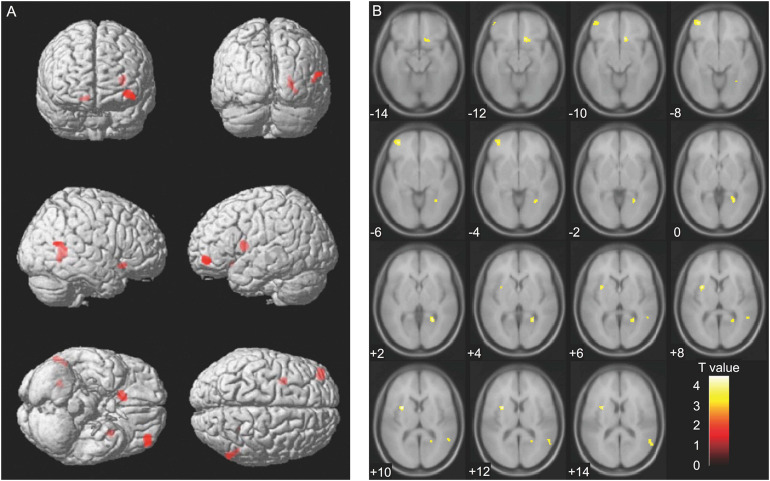
Voxel-based morphometry findings: Association between serum sTREM2 levels (Time 1) and brain volume (Time 2). Positive correlation between serum sTREM2 levels (Time 1) and brain volume (Time 2), as shown by multiple regression analysis. The threshold for statistics were set to *T* = 3.23 for the height threshold and *k* = 122 voxels for the extent threshold. Volumes of the important regions are shown in **(A)** whole brain images and **(B)** axial images. The *T*-value applies to **(B)** axial images.

We analyzed the correlation between serum sTREM2 levels (Time 1) and brain volume (Time 1, Time 2, and Time 1–Time 2 difference). However, analyses at the cluster level by applying multiple comparison corrections (family wise error; significance level, *P* < 0.05) showed no correlation between serum sTREM2 levels and volume of different brain regions. Therefore, an uncorrected analysis at the peak level was performed (significance level, *P* < 0.001). Results showed a positive correlation between serum sTREM2 level (Time 1) and volume of several brain regions (Time 2). These regions included the left putamen (coordinates −30, 5, 11), right lingual gyrus (coordinates 26, −53, 3), right olfactory cortex (coordinates 11, 21, −11), left middle frontal gyrus (coordinates −36, 50, −6), and right middle temporal gyrus (coordinates 57, −60, 14). The threshold for statistics were set to *T* = 3.23 for the height threshold and *k* = 122 voxels for the extent threshold. Table 3 shows the VBM findings. These findings are also shown in Figure 2 using standard brain MR images.

**TABLE 3:** Voxel-based morphometry (VBM) findings.

**Cluster-level**			**Peak-level**		**MNI coordinates**			
***P* FWE-corr**	***k*, Cluster size (voxels)**	***P* uncorr**	***T***	***P* uncorr**	***X* (mm)**	***Y* (mm)**	***Z* (mm)**	**Anatomical region**
0.709	148	0.253	4.52	<0.001	−30	5	11	Left putamen
0.601	198	0.188	4.35	<0.001	26	−53	3	Right lingual gyrus
0.767	122	0.298	4.23	<0.001	11	21	−11	Right olfactory cortex
0.543	227	0.160	4.17	<0.001	−36	50	−6	Left middle frontal gyrus
0.756	127	0.289	3.82	<0.001	57	−60	14	Right middle temporal gyrus

*Positive correlation between serum sTREM2 level (Time 1) and brain volume (Time 2) by multiple regression analysis. Height threshold, *T* = 3.23; Extent threshold, *k* = 122 voxels; Expected voxels per cluster, *k* = 121.917; Degrees of freedom = (1.0, 62.0); FWHM = 16.1, 15.9, and 15.2 mm; 10.7, 10.6, and 10.2 voxels; Volume, 1263188 = 374278 voxels = 281.0 resels; Voxel size, 1.5 mm × 1.5 mm × 1.5 mm (resel = 1157.29 voxels). FWE, family wise error; corr, corrected; uncorr, uncorrected; MNI, Montreal Neurological Institute; FWHM, full width at half maximum. Labels are marked using automated anatomical labeling.*

**FIGURE 3:**
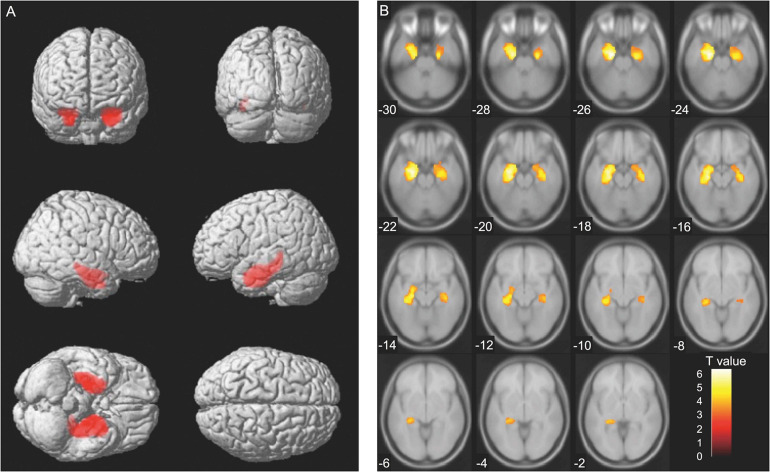
Voxel-based morphometry findings: Association between changes in clinical dementia rating (CDR) scores and brain volume (Time 2). Association between higher CDR scores and decreased brain volume (Time 2) evaluated using multiple regression analysis. The thresholds for statistics were set to *T* = 3.22 for the height threshold and *k* = 2684 voxels for the extent threshold. Significant clusters containing the left and the right hippocampus are shown in **(A)** whole brain images and **(B)** axial images. The *T*-value applies to **(B)** axial images.

Analyses at the cluster level by applying multiple comparison correction (family wise error; significance level, *P* < 0.05) showed no association between MMSE score difference (Time 1–Time 2 difference) and brain volume. However, higher CDR scores were associated with decreased volumes (Time 2) of the brain regions containing the left and right hippocampus. The thresholds for statistics were set to *T* = 3.22 for the height threshold and *k* = 2684 voxels for the extent threshold (Table 4). The VBM findings on the significant clusters containing the left and right hippocampus are shown in Figure 3 using standard brain MR images.

**TABLE 4:** Voxel-based morphometry (VBM) findings.

**Cluster-level**			**Peak-level**		**MNI coordinates**			
***P* FWE-corr**	***k*, Cluster size (voxels)**	***P* uncorr**	***T***	***P* uncorr**	***X* (mm)**	***Y* (mm)**	***Z* (mm)**	**Anatomical region**
<0.001	4658	<0.001	6.30	<0.001	−21	−12	−26	Left hippocampus
<0.001	2684	<0.001	5.37	<0.001	24	−15	−27	Right hippocampus

*Association between higher CDR scores and decreased brain volume (Time 2) by multiple regression analysis applying multiple comparison correction. These clusters contain the left and right hippocampus.*

*Height threshold, *T* = 3.22; Extent threshold, *k* = 2684 voxels; Expected voxels per cluster, *k* = 122.159;*

*Degrees of freedom = (1.0, 63.0); FWHM = 16.1, 15.9, and 15.2 mm; 10.7, 10.6, and 10.2 voxels;*

*Volume, 1263188 = 374278 voxels = 281.0 resels; Voxel size, 1.5 mm × 1.5 mm × 1.5 mm (resel = 1157.39 voxels).*

*FWE, family wise error; corr, corrected; uncorr, uncorrected; MNI, Montreal Neurological Institute; FWHM, full width at half maximum.*

*Labels are marked using automated anatomical labeling.*

### Additional Findings

We observed a decrease of both gray matter and total brain volume using paired *t*-test (*P* < 0.0001), by comparing Time 1 and Time 2. CDR (Time 1) scores were negatively associated with left hippocampus volume (Time 1) at the peak level (*P* < 0.001; the threshold for statistics were set to *T* = 3.22 for the height threshold and *k* = 123 voxels for the extent threshold). CDR (Time 2) scores were negatively associated with both hippocampus region’s volume (Time 2) at the cluster level (family wise error; *P* < 0.05). CDR (Time 1) scores were positively associated with volumes (Time 2) of only two regions, left precentral gyrus and superior temporal gyrus, at the peak level (*P* < 0.001; the threshold for statistics were set to *T* = 3.22 for the height threshold and *k* = 124 voxels for the extent threshold). CDR (Time 1) scores were positively associated with change of right hippocampus region’s volume (Time 1–Time 2 difference) at the cluster level (family wise error; *P* < 0.05). The change of CDR (Time 1–Time 2 difference) scores were negatively associated with change of whole brain regions’ volume (Time 1–Time 2 difference) at the cluster level (family wise error; *P* < 0.05). Additionally, we had focused 7 participants whose CDR score was either 0.5 or 1.0 at Time 2. In our sample, there were 6 participants with CDR of 0.5 and one participant with CDR of 1.0. As a demographic, we did not find a significant age difference between 7 participants and remaining 62 participants (75.42 ± 3.45 vs. 72.37 ± 4.32, *P* = 0.061). In addition, we did not find a significant difference of serum sTREM2 levels between 7 participants and remaining 62 participants (219.1 ± 175.5 pg/ml vs. 310.9 ± 291.7 pg/ml, *P* = 0.254). Lastly, analyses at the cluster level (family wise error; *P* < 0.05) showed no correlation between serum sTREM2 levels and volume of different brain regions in these 7 participants. Moreover, there was no association between serum sTREM2 levels (Time 2) and brain volume (Time 2) in any regions at the cluster level (family wise error; *P* < 0.05). Serum sTREM2 levels significantly increased from 301.6 ± 282.5 pg/ml (at Time 1) to 977.8 ± 963.5 pg/ml (at Time 2). The Wilcoxon signed-rank test showed a significant difference (*P* < 0.0001). When we used age as a covariate, serum sTREM2 difference between Time 2 and Time 1 was associated with changes in CDR (*P* = 0.016) but not in MMSE (*P* = 0.054). Lastly, we divided all participants to three groups according to baseline serum sTREM2 levels: low, medium, and high, respectively. We compared changes in serum sTREM2 levels among the three groups. As a result, there was no significant difference (*P* = 0.064), suggesting that relatively higher baseline serum sTREM2 levels did not increase later further.

Lastly, we analyzed the association between changes in serum sTREM2 levels (Time 1–Time 2 difference) and brain volume at Time 1, at Time 2, and Time 1–Time 2 difference, respectively. As a result, analyses at the cluster level (family wise error; *P* < 0.05) showed no association between changes in serum sTREM2 levels (Time 1–Time 2 difference) and brain volume (both at Time 1 and at Time 2). However, changes in serum sTREM2 levels (Time 1–Time 2 difference) were positively associated with changes in brain volume (Time 1–Time 2 difference) of only two regions containing the left frontal lobe and the left hippocampus (family wise error; *P* < 0.05). Thus, these suggest that the change of sTREM2 could serve as an indicator of the volume changes of both left frontal lobe and left hippocampus.

## Discussion

In this study, we focused on the correlation between serum sTREM2 levels and brain volume in people aged over 65 years. Analyses at the cluster level by applying multiple comparison corrections (family wise error; *P* < 0.05) showed no correlation between serum sTREM2 levels and brain volume, either cross-sectional or longitudinal. However, when analyzed uncorrected, we observed that the baseline serum sTREM2 levels correlate positively with the volume of several brain regions 7 years later at the peak level. In our previous studies using similar methods, we observed that serum oxytocin levels in the older adults were positively correlated with the future hippocampus and amygdala volumes (Orihashi et al., 2020). The hippocampus and amygdala are closely related to cognitive function. Whereas hippocampus is involved in regulating memory functions, amygdala is associated with regulating emotional functions, and both of these structures coordinate and interact (Scoville and Milner, 1957; Fink et al., 1996; Klüver and Bucy, 1997). In this study, we observed that MMSE scores decreased and CDR scores increased in participants 7 years after the baseline evaluation (Table 2). Moreover, higher CDR scores were also associated with decreased volume of the brain regions containing the left and right hippocampus (Table 4). Our results suggest that serum sTREM2 levels in older adults aged 65 years and above may not be significantly associated with later aging-related changes in the brain volume, especially in brain regions closely related to cognitive function.

Although previous studies have shown that CSF sTREM2 levels are higher in AD patients compared to healthy controls (Heslegrave et al., 2016; Piccio et al., 2016; Suárez-Calvet et al., 2016), others reported no difference between healthy controls and patients with AD or mild cognitive impairment (Henjum et al., 2016). Additionally, previous studies on sTREM2 levels in peripheral blood have also shown no difference between healthy controls and AD patients (Liu et al., 2018). Hu et al. reported an increased TREM2 expression at protein and mRNA levels on monocyte in AD, but plasma protein level was not significantly different in subjects with AD compared to controls (Hu et al., 2014). Moreover, there are also reports that high serum sTREM2 levels are associated with the development of dementia in the future (Ohara et al., 2019). These discrepancies in the results might be due to the characteristics of the participants. In the brain of AD patients, TREM2 expression may have a protective effect at an early stage (Ewers et al., 2019). However, in the later stages, there may be pathogenic effects through activation of the inflammatory response (Jay et al., 2017). A recent meta-analysis showed that sTREM2 level increases during the earlier course of AD development and is slightly attenuated at the dementia stage (Liu et al., 2018). There might be differences in sTREM2 expression and its clinical relevance between healthy individuals and patients at early and late stages of AD. Participants in our study were cognitively healthier at the baseline. This might be the reason that no association was observed between serum sTREM2 levels and future brain volume, especially in brain regions closely related to cognitive function. Additionally, the serum sTREM2 levels were divided into quartile categories (Q1 = 62.46–90.15 pg/ml, Q2 = 91.09–156.70 pg/ml, Q3 = 186.95–413.38 pg/ml, Q4 = 422.28–1415.8 pg/ml). We used linear mixed-effects models to estimate changes of averaged total brain volumes (A) and averaged MMSE scores (B) between Time 1 and Time 2 in each quartile category (Supplementary Figure 1). As a result, there were no interactions between serum sTRME2 levels and time, for total brain volume (*P* = 0.78) and MMSE (*P* = 0.40). Based on our results, serum sTREM2 levels in the older adults might not be considered as a biomarker for predicting a decline in future cognitive functions or brain volume. Recently, it has been suggested that higher CSF sTREM2 attenuates risk for cognitive decline and TREM2 may be protective against the development of AD (Franzmeier et al., 2020). Although measuring sTREM2 level in peripheral blood is less invasive and relatively easily, it might be necessary to analyze the relationship between the CSF sTREM2 levels and brain volume.

Our study has several major limitations. The evaluation of participants at Time 1 was conducted as part of a national survey for the prevalence of dementia in Japan. Importantly, MRI examinations were optional and were executed when necessary for further assessment of dementia or upon request of participants. Thus, in our study, selection of participants may be biased and the cohort may not reflect the characteristics of a general rural older adults. Although our study was aimed to evaluate serum sTREM2 levels and examine its relationship with brain volume using MRI, we did not measure biomarkers other than sTREM2, such as amyloid-beta, t-tau, and p-tau. Additionally, we used only MMSE and CDR to assess cognitive function. Moreover, compared to previous studies on serum sTREM2 levels (Ohara et al., 2019), our sample size was smaller. Our study was limited by the high number of individuals who dropped out during Time 1 and Time 2.

## Conclusion

Serum sTREM2 levels could not serve as an immune biomarker of aging-related volume changes in brain regions closely related to cognitive function in older adults aged 65 years and above.

## Data Availability Statement

The raw data supporting the conclusions of this article will be made available by the authors, without undue reservation.

## Ethics Statement

The studies involving human participants were reviewed and approved by the Ethics Committee of the Faculty of Medicine, Saga University, and all participants agreed to participate in the study according to the Declaration of Helsinki. The patients/participants provided their written informed consent to participate in this study.

## Author Contributions

RO, YM, YI, SY, and AM designed the study. RO, YM, and YI acquired the data. RO analyzed the data and drafted the manuscript. YM edited the manuscript. All authors contributed to the article and approved the submitted version.

## Conflict of Interest

The authors declare that the research was conducted in the absence of any commercial or financial relationships that could be construed as a potential conflict of interest.

## Abbreviations

AD: Alzheimer’s disease
BMI: body mass index
CDR: clinical dementia rating
CSF: cerebrospinal fluid
DARTEL: diffeomorphic anatomical registration through exponentiated lie algebra
FWE: family wise error
FWHM: full width at half maximum
hs-CRP: high-sensitivity C-reactive protein
MMSE: Mini-Mental State Examination
MNI: Montreal Neurological Institute
SPM: Statistical Parametric Mapping
TREM2: triggering receptor expressed on myeloid cells 2
VBM: voxel-based morphometry.
