# Supplementary material for: Association Between sTREM2, an Immune Biomarker of Microglial Activation, and Aging-Related Brain Volume Changes in Community-Dwelling Older Adults: A 7-Year Follow-Up Study
Source: Front Aging Neurosci. 2021 Apr 26;13:665612. doi: 10.3389/fnagi.2021.665612 (PMC8109266; doi:10.3389/fnagi.2021.665612)
Supplement: Supplementary file 1 [file Table_1.DOCX]

***Supplementary Material***

**Supplementary Figure 1**

**Linear mixed-effects models to estimate changes of total brain volume and MMSE scores between Time 1 and Time 2 in quartile categories according to serum sTREM2 levels**

The serum sTREM2 levels were divided into quartile categories (Q1 = 62.46–90.15 pg/ml, Q2 = 91.09–156.70 pg/ml, Q3 = 186.95–413.38 pg/ml, Q4 = 422.28–1415.8 pg/ml). We used linear mixed-effects models to estimate changes of averaged total brain volumes (A) and averaged MMSE scores (B) between Time 1 and Time 2 in each quartile category using a statistical package (JMP 14.2.0). There was no significant difference between changes in total brain volume and serum sTREM2 levels (P = 0.14), but was a significant difference with time (P < 0.0001). Similarly, there was no significant difference between changes in MMSE and serum sTREM2 levels (P = 0.25), but was a significant difference with time (P < 0.0001). In addition, there were no interactions between serum sTRME2 levels and time, for total brain volume (P = 0.78) and MMSE (P = 0.40).
